# Sex-dependent biobehavioural responses to transgenerational maternal stress: evidence of stress resilience and vulnerability in the F3 generation

**DOI:** 10.1093/eep/dvaf031

**Published:** 2025-11-06

**Authors:** Jamshid Faraji, Nicola Schatz, Stephanie King, Nasrin Soltanpour, Gerlinde A S Metz

**Affiliations:** Canadian Centre for Behavioural Neuroscience, Department of Neuroscience, University of Lethbridge, Lethbridge, Alberta T1K 3M4, Canada; Canadian Centre for Behavioural Neuroscience, Department of Neuroscience, University of Lethbridge, Lethbridge, Alberta T1K 3M4, Canada; Canadian Centre for Behavioural Neuroscience, Department of Neuroscience, University of Lethbridge, Lethbridge, Alberta T1K 3M4, Canada; Canadian Centre for Behavioural Neuroscience, Department of Neuroscience, University of Lethbridge, Lethbridge, Alberta T1K 3M4, Canada; Canadian Centre for Behavioural Neuroscience, Department of Neuroscience, University of Lethbridge, Lethbridge, Alberta T1K 3M4, Canada; Southern Alberta Genome Sciences Centre, University of Lethbridge, Lethbridge, Alberta T1K 3M4, Canada

**Keywords:** transgenerational prenatal maternal stress, ancestral stress, pregnancy, sex differences, HPA axis, epigenetics, anxiety-like behaviour, cytokines, social isolation, restraint stress

## Abstract

Maternal prenatal stress can determine stress resilience and vulnerability of future generations. However, the extent to which the biological sex of the descendants determines the response to ancestral stress is not fully understood. In this study, neurohormonal responses and exploratory and anxiety-like behaviours were examined in third-generation (F3) male and female rats born to non-stressed and transgenerationally stressed lineages, where maternal stress was induced only in pregnant females of the ancestral F0 generation. While ancestral stress in F3 females did not alter hypothalamic–pituitary–adrenal (HPA) axis activity, F3 males born to F0 stressed mothers exhibited HPA axis hyperactivity compared to non-stressed males. By contrast, females revealed significantly higher corticosterone levels than males. Moreover, ancestral stress elevated concentrations of the cytokines interleukin-1β (IL-1β) and IL-10 exclusively in females. Ancestral maternal stress also produced task-specific differences in depressive- and anxiety-like symptoms in the F3 generation, particularly in females. Specifically, F3 female behaviour within the open field and elevated plus maze tasks was more affected by ancestral maternal stress than that of F3 males. Supported by correlational analysis, the findings demonstrate that F3 female offspring are more sensitive than males to the neuroimmunological and behavioural impacts of maternal prenatal stress, despite the absence of elevated HPA axis activity. In contrast, males primarily responded with HPA axis activity upregulation, which compounded effects on their behavioural profile. The present study supports the notion that maternal stress, across generations, is likely to epigenetically programme sex-specific behavioural, physiological, and immunological phenotypes in remote offspring, with particular vulnerability in females.

## Introduction

A family history of adverse experiences can be linked to heightened vulnerability to neuropsychiatric disorders [1, 2]. Pregnancy is a critical time when the foetal brain is particularly vulnerable to both organizing and activating influences of hormones and molecular factors. Thus, maternal stress during pregnancy may have long-term adverse consequences on foetal endocrine function involving the hypothalamic–pituitary–adrenal (HPA) axis [3, 4]. For instance, prenatal stress may re-programme HPA axis function and result in increased sensitivity to stress in later life [5, 6] and future generations [7, 8]. Recent findings have shown that both adaptive and maladaptive functional and anatomical consequences of prenatal stress may propagate to at least the third (F3) or fourth (F4) generations [9–12]. Therefore, individuals whose ancestors were exposed to stress may experience a higher risk of mental and physical health problems [10, 11, 13]. Neuropsychiatric complications of inter- and transgenerational stress may include low mood, anhedonia, and loss of motivation [1, 14] or increased reactivity to stress and depression-like states [15–17].

Responses to stress, either immediate or ancestral, can also be modulated by sex-related influences. As an inherent biological variable, biological sex determines a wide range of physiological and behavioural stress responses in humans and non-human animals [18, 19]. In fact, HPA axis activity as the key adaptive neuroendocrine response to stressors is characterized by prominent sex differences. For example, female rodents appear to initiate HPA axis activity more rapidly in response to stress signals and produce a greater output of stress hormones relative to males [20]. When compared to males, females also display higher baseline glucocorticoid [corticosterone (CORT) in rodents] levels and higher absolute CORT concentrations during stress [20, 21]. Behaviourally, skilled movements in female rats seem to adapt more rapidly to daily stress and recover faster from stress than males [22]. When chronic stress exposure is inevitable, females seem to reveal more flexible and adaptive behavioural responses than males [23]. Notably, sex differences in HPA function are evident already early in life [24] and contribute to distinct biobehavioural responses in adulthood. Thus, sex differences explain much of the variability in the responses to immediate and ancestral stress.

Aside from sex differences in HPA axis function, the immune system, which acts in close dialogue with the neuroendocrine system [25, 26], also exhibits significant sex differences [27]. These sex disparities include differential expression of cytokines, which are signalling proteins produced by immune cells [28–30]. Stressful experiences may trigger emotion-related (e.g. depression- and anxiety-like) behaviours in rodents by inducing a pro-inflammatory response in both central neural networks linked to emotional processing and peripheral immune units [31, 32]. Cytokines, such as interleukins and tumour necrosis factors, have been shown to mediate stress-induced behavioural phenotypes by modulating neural circuits associated with mood regulation. Specifically, elevated pro-inflammatory cytokine levels in response to stress can exacerbate anxiety-like behaviours and impair coping mechanisms in rodents [33]. Importantly, exposure to early life stress can lead to long-lasting alterations in the immune system and neuroinflammatory state, thus increasing the risk of developing psychiatric disorders in later life [34]. Cytokines, particularly pro-inflammatory interleukin-1β (IL-1β) and tumour necrosis factor-alpha (TNF-α), have been implicated in heightened depressive-like and anxiety-like behaviours [32, 35]. Stress-related increases in these cytokines influence neural circuits involved in mood regulation, such as the hippocampus and prefrontal cortex, contributing to maladaptive behavioural phenotypes [30, 36].

We have previously shown in rats that prenatal experiences in the F0 generation can produce new biobehavioural phenotypes that propagate to subsequent generations [7, 37]. Using a maternal transgenerational prenatal stress (MTPS) protocol, we also showed that prenatal stress promotes psychomotor retardation and alters HPA axis activity in F4 generation rats [38]. In the present study, we hypothesized that F0 maternal gestational psychosocial stress influences stress adaptability in the remote F3 progeny in a sex-dependent manner. Pregnant F0 generation dams were exposed to restraint and social isolation (SI) stress during late gestation. In the female lineage, the F3 generation provides the first offspring programmed by transgenerational epigenetic inheritance [39, 40]. Thus, the F1–F2 generation offspring were left undisturbed, and their F3 male and female descendants were examined for biobehavioural responses to the ancestral maternal stress. The findings support the notion that ancestral stress exposure may generate new, sex-specific biobehavioural phenotypes that can be transmitted to the remote progeny. This study underscores the critical role of sex in modulating stress responsiveness and highlights the importance of addressing ancestral stress to better resolve current health disparities.

## Results

### Maternal stress alters sex-dependent locomotion and affective behaviour in open field exploration in the F3 generation

**Figure 1.**
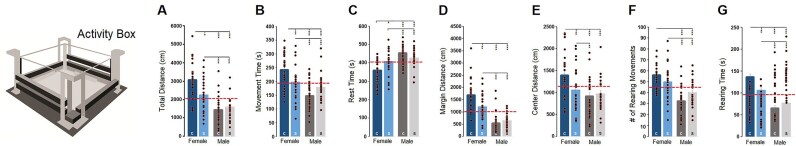
Open field behaviours in the F3 generation. On the left, a representative illustration of the automated open field test. Seven measures of exploratory behaviour in the open field (panels A–G) in F3 female and male rats revealed a sex-dependent response to the maternal prenatal stress (*n* = 65–90/group). Asterisks indicate significance: *P ≤ .05; **P ≤ .01, ***P ≤ .001, *post hoc* Tukey. The dotted lines indicate the mean score of each parameter in different groups.

Multivariate analysis of variance (MANOVA) showed a main effect of group (Table 1) in seven behavioural measures of open field exploration (*total travel distance, F*_3,326_ = 58.87, *P* ≤ .001, *η*^2^ = 0.35; *movement time, F*_3,326_ = 46.10, *P* ≤ .001, *η*^2^ = 0.29; *rest time, F*_3,326_ = 46.09, *P* ≤ .001, *η*^2^ = 0.29; *margin distance, F*_3,326_ = 76.15, *P* ≤ .001, *η*^2^ = 0.41; *centre distance, F*_3,326_ = 20.53, *P* ≤ .001, *η*^2^ = 0.15; *number of rearings, F*_3,326_ = 55.22, *P* ≤ .001, *η*^2^ = 0.33; *rearing time, F*_3,326_ = 49.22, *P* ≤ .001, *η*^2^ = 0.31) where all groups travelled significantly less in the open field compared to control females. Moreover, control females also displayed higher movement time and lower rest time, higher margin and centre distance along with higher rearing time than other groups (all *P* ≤ .05; *post hoc* Tukey, Fig. 1). Also, stressed females indicated significantly lower locomotion than control females in most open field parameters as well as higher rate of exploration within the task when compared with both control and stressed males (Fig. 1A–G). Further, males in both groups displayed a noticeably different profile of exploratory behaviour than females where the distance travelled by both groups over the 10-minute test session was significantly shorter, the movement time was lower, the rest time was higher, and the rearing time was lower than among females (all *P* ≤ .05), even though there were no differences between stressed and control males (all *P* ≥ .05). Therefore, transgenerational stress exposure in F3 animals was associated with a sex-dependent psychomotor pattern through which stressed females displayed reduced exploratory behaviour in the open field when compared with F3 control females along with increased activity relative to F3 control and stressed males.

**Table 1.** Summary of sex-dependent differences in affective behaviour in open field

Outcome measure	Test statistic	*P*-value	Effect size
Main effect of group			
*Total travel distance*	*F*_3,326_ = 58.87	.001	*η* ^2^ = 0.35 (MANOVA)
*Movement time*	*F*_3,326_ = 46.10	.001	*η* ^2^ = 0.29 (MANOVA)
*Rest time*	*F*_3,326_ = 46.09	.001	*η* ^2^ = 0.29 (MANOVA)
*Margin distance*	*F*_3,326_ = 76.15	.001	*η* ^2^ = 0.41 (MANOVA)
*Centre distance*	*F*_3,326_ = 20.53	.001	*η* ^2^ = 0.15 (MANOVA)
*Number of rearing*s	*F*_3,326_ = 55.22	.001	*η* ^2^ = 0.33 (MANOVA)
*Rearing time*	*F*_3,326_ = 49.22	.001	*η* ^2^ = 0.31 (MANOVA)

### Maternal stress alters anxiety-like behaviours in elevated plus maze especially in female F3 offspring

**Figure 2.**
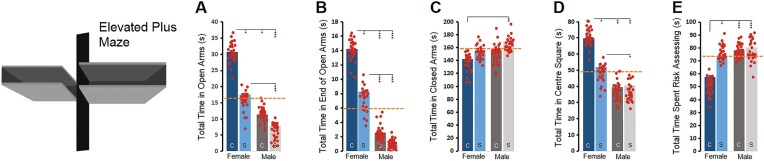
Performance in the elevated plus maze in the F3 generation. On the left, a representative illustration of the elevated plus maze. The NNN**N** females evidently indicated lower anxiety-like behaviour in the EPM than all other groups, including their SNN**N** female counterparts. However, SNN**N** female rats displayed patterns of behavioural changes influenced by maternal prenatal stress that were not as evident as changes observed in the activity box (*n* = 24–28/group). Asterisks indicate significance: *P ≤ .05, **P ≤ .01, ***P ≤ .001, *post hoc* Tukey. The dotted lines indicate the mean score of each parameter in different groups.

Anxiety-like behaviour in F3 generation rats was examined using the elevated plus maze (EPM) (Fig. 2, left panel). A significant main effect of group (Table 2) was found in five elemental measures of anxiety-like behaviour in the EPM (total time in open arms, *F*_3,313_ = 9.68, *P* ≤ .001, *η*^2^ = 0.08; total time in end of open arms, *F*_3,313_ = 19.56, *P* ≤ .001, *η*^2^ = 0.15; total time in closed arms, *F*_3,313_ = 4.55, *P* ≤ .004, *η*^2^ = 0.04; total time in centre square, *F*_3,313_ = 10.41, *P* ≤ .001, *η*^2^ = 0.09; total time spent risk assessing, *F*_3,313_ = 10.22, *P* ≤ .005, *η*^2^ = 0.08; MANOVA; Fig. 2A–E), suggesting that F3 control females (*n* = 86) spent significantly more time than other groups in the open arms and the end of open arms along with less time in closed arms (all *P* ≤ .05; *post hoc* Tukey). Maternal prenatal stress, however, induced enhanced anxiety-related responses in F3 stressed females (*n* = 78) where they tended to spend less time in open arms (*P* ≤ .05; *post hoc* Tukey; Fig. 2A) and more time in closed arms when compared to their female and male counterparts. Both F3 groups of males, on the other hand, displayed significantly reduced EPM-searching behaviour with less time spent in open arms and more time in closed arms relative to control and stressed females. Total time spent in centre square and risk assessing behaviour in the EPM was also influenced by sex and ancestral stress in males relative to females (Fig. 2D–E). There were no differences between stressed and control F3 males (*n* = 70 and *n* = 83; all *P* ≥ .05). Like open field, it appears that ancestral maternal prenatal exposure to stress had a higher impact on anxiety-like behaviour in F3 females. However, unlike the open field, stressed females revealed a profile of anxiety-like behaviours in the EPM, which was more comparable to their male counterparts.

**Table 2.** Summary of sex-dependent differences in anxiety-like behaviours in elevated plus maze

Outcome measure	Test statistic	*P*-value	Effect size
Main effect of group			
*Total time in open arms*	*F*_3,313_ = 9.68	.001	*η* ^2^ = 0.08 (MANOVA)
*Total time in end of open arms*	*F*_3,313_ = 19.56	.001	*η* ^2^ = 0.15 (MANOVA)
*Total in closed arms*	*F*_3,313_ = 4.55	.004	*η* ^2^ = 0.04 (MANOVA)
*Total time centre square*	*F*_3,313_ = 10.41	.001	*η* ^2^ = 0.09 (MANOVA)
*Total time spent risk assessing*	*F*_3,313_ = 10.22	.005	*η* ^2^ = 0.08 (MANOVA)

### Maternal stress alters circulating plasma CORT and cytokine concentrations especially in F3 female offspring

**Figure 3.**
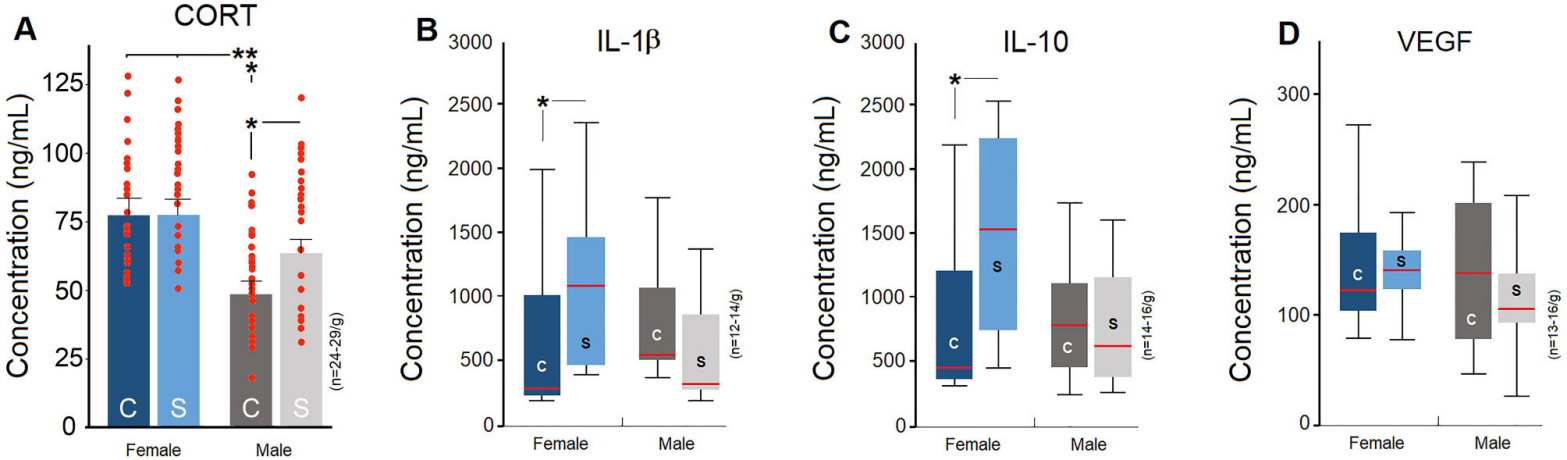
Circulating levels of CORT and cytokines. (A) Females from ancestrally non-stressed (*n* = 26) and stressed (*n* = 24) lineages similarly displayed higher levels of CORT compared to their male counterparts. However, male rats who were exposed to maternal prenatal stress (*n* = 24) showed an exacerbated CORT response when compared to males from the non-stressed lineage (*n* = 29). (B–D) Plasma cytokine levels, on the other hand, showed upregulated IL-1β and IL-10 in stressed females, whereas VEGF expression was not affected by the ancestral maternal stress (*n* = 12–16/g). Asterisks indicate significance: *P ≤ .05, ***P ≤ .001, *post hoc* Tukey and Kruskal–Wallis test followed by pairwise comparison.

Figure 3A illustrates CORT levels in F3 control and stressed female and male rats. A representative sample of animals from each group (*n* = 24–29) was randomly selected for CORT assays. A significant main effect of group (*F*_3,98_ = 12.08, *P* ≤ .001, *η*^2^ = 0.27) was shown by *univariate* analysis of variance (UANOVA) where F3 females in both groups (Table 3) showed higher levels of CORT than both groups of F3 males. While there were no differences between stressed and control females (*n* = 26 and *n* = 24; *P* ≥ .05), stressed males (*n* = 24) showed significantly higher levels of CORT than control males (*n* = 29; *P* ≤ .05, *post hoc* Tukey), suggesting that F3 stressed male rats from the ancestrally stressed lineage had more exaggerated HPA axis responses than males who were not exposed to ancestral prenatal stress. Further, pro- and anti-inflammatory responses to maternal stress were measured via changes in circulating cytokine concentrations (Fig. 3B–D). There was a statistically significant group difference (*H_3_* = 8.89, *P* ≤ .05; *n* = 12–14/group) with a mean rank IL-1β level of 19.86 for control females, 34.89 for stressed females, 33.13 for control males, and 23.04 for stressed males. Also, there was a significant difference between groups in terms of changes in IL-10 (*H_3_* = 9.77, *P* ≤ .05; *n* = 14–16/group) where mean rank of IL-10 levels was 25.04 for control females, 43.09 for stressed females, 30.78 for control males, and 26.28 for stressed males (Table 4). Multiple comparisons showed differences between control and stressed females in IL-1β and IL-10 concentrations. However, the Kruskal–Wallis *H* test showed no significant differences between groups in vascular endothelial growth factor (VEGF) levels (*P* ≥ .35; *n* = 13–16/group). Thus, it appears that ancestral maternal prenatal stress may contribute to variations in neuroinflammatory mediators in remote female progeny.

**Table 3.** Summary of group differences in CORT levels (*n* = 24–28/group)

Outcome measure	Test statistic	*P*-value	Effect size
Main effect of group	*F*_3,98_ = 12.08	.001	*η* ^2^ = 0.27 (UANOVA)
Fc,s ˃ Mc		.001	(Tukey, Honestly Significant Difference [HSD])
Mc ˂ Ms		.04	(Tukey, HSD)

**Table 4.** Summary of group differences in cytokine levels (Kruskal–Wallis test)

Cytokine	IL-1β	Cytokine	IL-10
Total *n*	55	Total *n*	62
Test statistics	8.898	Test statistics	9.770
Degrees of freedom	3	Degrees of freedom	3
Asymptotic sig.	.031	Asymptotic sig.	.021

### Correlational analysis

Correlation coefficients were calculated to examine the correlation between the CORT levels and behavioural measures in females and males.

**Figure 4.**
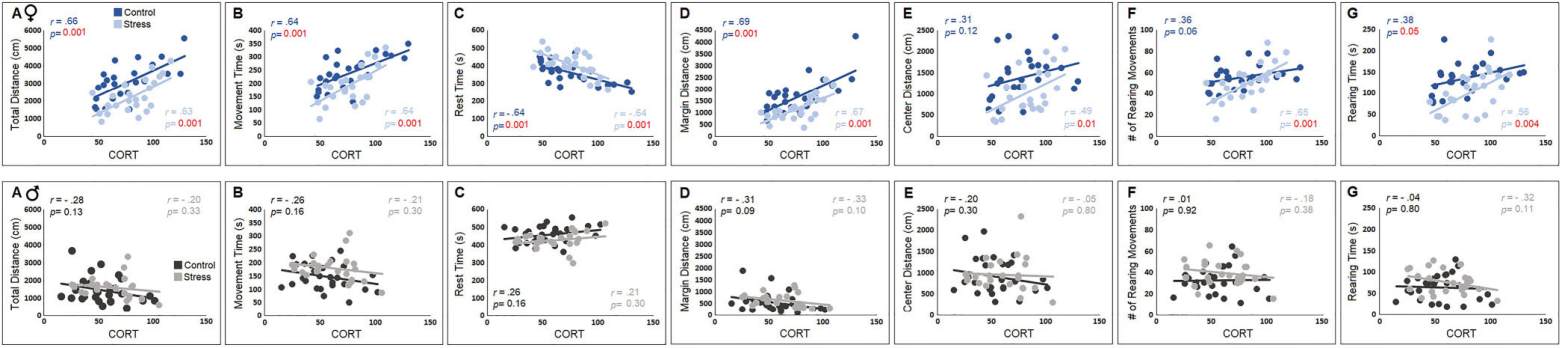
Correlation between locomotor behaviour in open field and CORT levels in females and males. (Top row AȓG) A significant correlation was found between most behavioural parameters in open field and CORT values in females. (Bottom row A–G) There was no significant correlation between these variables in males. Females from the stress lineage showed stronger correlation than non-stressed females. The CORT levels only in stressed females predict alterations in locomotor behaviour.

*Open field*: Correlational analysis revealed a significant relationship between plasma CORT and changes in exploratory behaviour within the automated open field. It was shown that enhanced CORT levels were significantly associated with increased levels of locomotion in both control and stressed F3 females (*total distance* [**C:** *r_(23)_* = 0.66, *P* ≤ .001; **S:** *r_(20)_* = 0.63, *P* ≤ .001], *movement time* [**C:** *r_(23)_* = 0.64, *P* ≤ .001; **S:** *r_(20)_* = 0.64, *P* ≤ .001], *rest time* [**C:** *r_(23)_* = -0.64, *P* ≤ .001; **S:** *r_(20)_* = -0.64, *P* ≤ .001], *margin distance* [**C:** *r_(23)_* = 0.69, *P* ≤ .001; **S:** *r_(20)_* = 0.67, *P* ≤ .001], *centre distance* [**C:** *r_(23)_* = 0.31, *P* ≥ .12; **S:** *r_(20)_* = 0.49, *P* ≤ .01], *number of rearing movement* [**C:** *r_(23)_* = 0.36, *P* ≥ .06; **S:** *r_(20)_* = 0.65, *P* ≤ .001], *rearing time* [**C:** *r_(23)_* = 0.38, *P* ≤ .05; **S:** *r_(20)_* = 0.56, *P* ≤ .004]; Pearson’s correlation coefficient; Fig. 4A–G, top). Further, while females’ exploratory behaviour in the open field was remarkably associated with increased HPA axis and CORT responses, it was indicated (Fig. 4A–G, down) that CORT levels in response to ancestral maternal prenatal stress in F3 males could not predict behavioural responses in the task (*n* = 24 and 29/group, all *P* ≥ .05, Pearson’s correlation coefficient and simple linear regression). Accordingly, although measures of CORT levels alone may not be sufficient to decisively explain the existing profile of locomotion, the enhanced CORT response observed in the present study could robustly predict higher locomotion only in females, regardless of their experimental conditions within the open field compared to their male counterparts.

**Figure 5.**
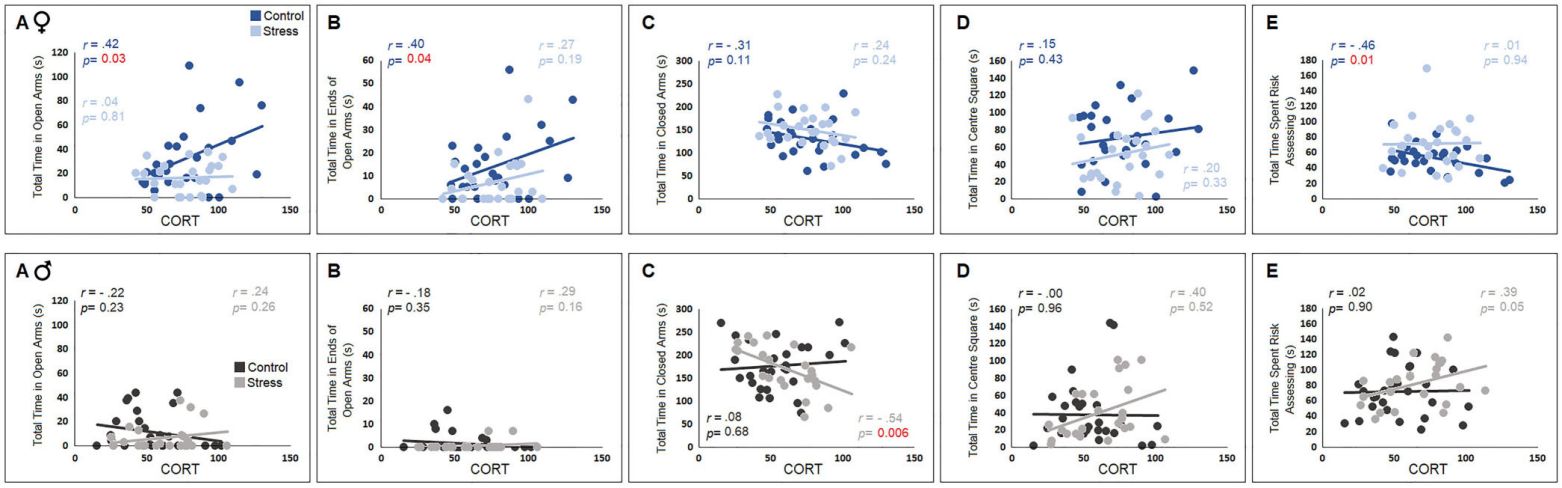
Correlation between anxiety-like behaviours in elevated plus maze and CORT levels in females and males. (Top row A-G) While there was a significant correlation between some EPM measures for anxiety-like behaviour in control females, stressed females indicated no significant correlation. (Bottom row A-G) In males, on the other hand, there was a negative correlation between total time spent in the closed arms and CORT levels only in the stressed group. The CORT levels failed to predict changes in anxiety-like behaviours.

*Elevated plus maze*: Figure 5 illustrates correlations between CORT levels and measures of the anxiety-like behaviours in the EPM. Elevated CORT levels were associated with the time spent in open arms (*r_(22)_* = 0.42, *P* ≤ .03), and the time in the end of open arms (*r_(22)_* = 0.40, *P* ≤ .04), as well as the total time spent in risk assessing (*r_(22)_* = -0.46, *P* ≤ .01, Pearson’s correlation coefficient) in control females only (Fig. 5A–E, top). Thus, CORT levels can reliably predict some aspects of anxiety-related behaviours only in females from non-stressed ancestral lineage (time spent in open arms, time in the end of open arms, time spending risk assessing; simple linear regression). In males, however, the correlation between CORT responses to the ancestral stress and EPM measures was significant for the stressed group only in the time spent in closed arms (*r_(27)_* = -0.54, *P* ≤ .006, Pearson’s correlation coefficient, Fig. 5A–E, down), indicating that increased plasma CORT can predict reduced time spent in the closed arms by ancestrally stressed F3 males (simple linear regression). Overall, it appears that changes in HPA axis activity by ancestral stress can predict behavioural alterations in F3 descendants in a sex-dependent and task-specific manner.

## Discussion

Exposure to gestational stress is a key determinant of the stress response later in life and across generations. The major findings of this study show that maternal gestational stress (i) can propagate transgenerationally across three subsequent generations; (ii) can generate a phenotype that is vulnerable to neuroimmunological challenges and depressive-like states, especially in females; and (iii) is distinctively associated with dysregulation of HPA axis activity and psychomotor inhibition in F3 animals in a sex-dependent and task-specific manner. The present study therefore provides support for our previous reports that ancestral maternal prenatal stress impacts subsequent generations through producing new behavioural and immunological phenotypes [9, 41]. Together, these findings suggest that ancestral stress challenges normal adaptive biobehavioural and immunological mechanisms required for lifetime health.

HPA axis hyperactivity is a hallmark of the response to both transient and repeated stressful experiences in rodents [42], with distinct neuroendocrine responses to stress via the HPA axis among females versus males [20]. The present study focused on HPA and cytokine responses to ancestral prenatal stress because dysregulation of the HPA axis and neuroinflammatory processes is a critical factor in disease pathogenesis, and their regulation is strongly influenced by epigenetic mechanisms [43, 44]. For example, SI stress elicits a heightened stress response, primarily through HPA axis dysregulation [41], and SI in the F4 generation of ancestrally stressed rats may downregulate HPA axis activity [7]. More importantly, the immune system that operates in close interaction with the neuroendocrine system, including the HPA axis, also exhibits marked sex differences [27]. Maternal stress exposure in the F0 generation dysregulated HPA axis responsiveness and resulted in increased levels of basal circulating CORT levels in third-generation males, but had little impact on these measures in females. Elevated levels of glucocorticoids represent a widely accepted neurohormonal correlate of stress response [45] that can be adaptive or maladaptive. The adaptive aspect of the stress response, which is associated with activation of the HPA axis, is fundamental for an organism’s survival. However, the extent to which maladaptive or harmful effects of transgenerational stress can influence the degree of resilience or stress sensitivity in offspring, especially in generally less resilient males, is not yet fully understood. These profound neuroendocrine changes associated with ancestral maternal stress therefore may have adverse consequences for mental and physical health outcomes in future generations.

The clinical concept of inter- and transgenerational transmission of stress and trauma [46–48] is supported by empirical data [7, 48–50] refers to a vital physiological and epigenetic process via which a traumatic memory propagates from the parental generation to their offspring. This mechanism primarily serves to enable rapid and functionally meaningful adaptation of behavioural, immunological, and metabolic responses to a stressful environment [51]. However, an epigenetic memory of severe or cumulative stress may also threaten the adaptive value of transgenerational reprogramming by increased physiological ‘wear and tear’, which may result in pathological risks, such as psychopathologies or non-communicable diseases [49, 52–56]. These may occur in part as a consequence of disrupted *in utero* neurodevelopment [50], poor maternal care [8], or through impaired learning and memory networks centred on the amygdala and hippocampus [57]. As seen here and in our previous studies [7, 8, 38], such effects may also be mediated by stress-induced HPA axis dysregulation and glucocorticoid-related challenges, which may involve altered regulation of brain-derived neurotrophic factor (BDNF) [17, 58, 59]. BDNF not only promotes brain development and aging but also represents a vital marker of lifetime stress resilience that promotes adaptive synaptic plasticity and coping behaviours during stressful experiences [60].

Here, we report that despite the males’ HPA axis vulnerability to ancestral prenatal stress, their locomotion and exploratory behaviours remained unchanged. In contrast, the impact of ancestral stress on F3 females appears paradoxical; although their HPA axis activity remains unaltered, they show increased susceptibility to behavioural disruptions. The inhibitory effects on exploratory behaviours in stressed females may be linked to high emotionality (anxiety and fear), fatigue, and behavioural despair [7, 61]. CORT levels were also differentially associated with behavioural measures in females and males, further supporting the notion of sex-dependent HPA axis function in response to stress. Accordingly, it seems unjustified to define a stress state and/or a stress-induced biobehavioural phenotype based on only the HPA axis hyper- and hyporeactivity [62]. Our previous findings have indicated that physiological responses to stress, such as elevated body temperature, may occur independently of elevated CORT levels, particularly in females [28, 42]. Moreover, as a key neuroendocrine circuit, the HPA axis modulates hormonal responses to internal and external challenges that are typically reflected in behavioural changes. Hence, stressed organisms may experience high emotionality, which may in turn aggravate normal behavioural responses.

The present data suggest a prominent sex-dependent influence of ancestral stress on cytokine production and signalling. This may explain to some extent why parental psychosocial distress may render remote descendants more susceptible to neuroinflammatory responses to autoimmune and inflammatory diseases [10, 11, 52]. Importantly, the expression of cytokines such as IL-1β and IL-10 follows a sex-dependent trajectory of anti- and pro-inflammatory responses to stressful experiences [63]. These differences may be modulated by sex hormones and their interaction with immune signalling pathways, with oestrogen shown to exert protective, anti-inflammatory effects, especially in females. Moreover, stress exposure in males more reliably correlates with long-term increases in peripheral inflammation, which may contribute to greater vulnerability to certain stress-related disorders [64]. Epigenetic changes may also contribute to the sex-dependent differences in cytokine responses to stress. For instance, stress-induced changes in DNA methylation at cytokine gene promoters, such as IL-1β and IL-10, differ by sex, with females often showing greater epigenetic flexibility in regulatory regions [65]. Oestrogen can further modulate these epigenetic marks, leading to enhanced anti-inflammatory cytokine expression in females compared to males [66]. Also, sex-specific histone modifications in immune-related genes have been observed following early-life or chronic stress that may suggest a persistent, sex-biased tuning of inflammatory responses [67, 68]. Sex differences in cytokine responses to stress, therefore, may be rooted in dynamic, lifelong interactions between hormonal milieus and epigenetic programming.

Here, F3 stressed females displayed an augmented production of pro-inflammatory cytokine IL-1β and anti-inflammatory cytokine IL-10, but no deficiency to produce VEGF. Behaviourally, elevated IL-1β secretion is associated with anxiety, fear, and exacerbated emotional responses towards acute and chronic stress [69–72]. The female-biased overproduction of IL-1β and IL-10 in response to maternal stress, however, depicts an alternative picture of parental stress influences on remote progeny, which involve two contradictory aspects of cytokine expression. First, the dysregulated IL-1β secretion in stressed females was not associated with HPA axis hyperactivity in terms of elevated CORT levels, yet IL-1β overexpression is known to activate the HPA axis [73]. This discrepancy may be partially related to estrous cycle stages in females that were not determined in the present experiment. Although for most traits, estrous cycles in rodent research do not need to be controlled [74], the present findings cannot rule out that the estrous cycle may confound both CORT levels and IL-1β dysregulation in females. Because IL-1β acts as the principal suppressor of the hypothalamic–pituitary–gonadal (HPG) axis, it may also play a key role in inhibiting reproductive functions, thus interrupting the mutual complex dialogue between the HPA and HPG systems [75], especially through its potent modulatory effects on the catecholamine norepinephrine [73]. Indeed, the mechanisms through which transgenerational mother-to-daughter transmission of stressful experiences may lead to imbalanced expression of glucocorticoids and cytokines still require further investigation.

The second contradictory point refers to the functional connections between IL-10 as a potent immunosuppressive cytokine and growth factors such as VEGF. It is well known that VEGF signalling contributes to neurogenesis [76] and angiogenesis [77], especially in pathological processes that form blood vessels in cancerous tumours. VEGF biological function is tightly regulated by IL-10, which is known as anti-angiogenic protein [78, 79]. Thus, IL-10 upregulation by stress usually occurs in the presence of downregulation of VEGF, and *vice versa*. Interestingly, immediate psychological stress can result in overexpression of IL-10 cytokines [80], which arguably represents a potential neuroinflammatory pathway to the stress-induced immunosuppression. Here, however, we observed increased IL-10 signalling in F3 stressed females while VEGF remained unchanged. This might be related to compensatory responses during the rather chronic nature of ancestral stress. Thus, the sex-specific relationships between cytokine signalling and HPA axis regulation and psychomotor disruption still remain to be further investigated.

Further consideration needs to be given to the types of behavioural assessments used. The heterogenous pattern of behavioural responses in the present study indicates that not only neurohormonal characteristics, but also task demands and conditions may confound sex differences in response to ancestral stress. Recently, we have shown that experimental procedures and the presence of human experimenters are significant determinants of stress responses in a sexually dimorphic manner [81]. Thus, beyond intrinsic disparities between females and males that may impact experimental results, it appears that extrinsic variables such as structural differences between tasks (e.g. physical features, task demands, and procedures) also influence behavioural consistency in females and males. This is specifically important when investigating motivational correlates of exploratory behaviour in females and males within the open field task. Because an open field is an anxiogenic task by nature [82] and open field activity provides the primary indicator of emotionality, the task demands may play a key role in behavioural variations along with sex-related influences.

The effects of prenatal stress in the present study were reflected in behavioural changes only in remote female offspring. Thus, ancestral maternal stress across generations may limit behavioural flexibility (e.g. intensified depressive-like behaviours) through the female lineage (mother-to-daughter), despite an undisturbed HPA axis function. The transgenerational transmission of stress from the gestationally traumatized F0 generation to remote offspring may occur in a sexually dimorphic manner and via the female lineage [37, 83]. Nevertheless, prenatal programming of HPA axis properties itself may partly explain the large behavioural variability in response to immediate and ancestral stressors among females and males. Generally, the disruptive neurohormonal and behavioural consequences of prenatal stress seem to be more prominent in males than in females [84]. The differential impact of stress on behavioural outcomes in females and males, however, has been linked to sex-specific epigenetic regulation of stress resilience [85] and differential endocrine and neurotransmitter responses [86]. It is noteworthy that glucocorticoid levels remain elevated for longer in stressed females [23], suggesting an increased stress reactivity in females with potentially adaptive benefits, and faster recovery in specific behaviours [22]. The mother-to-daughter lineage of transmission seen in behavioural alterations without HPA axis responses in females as opposed to behavioural insensitivity in males in the presence of HPA axis hyperactivity arguably reflects sex-dependent epigenetic programming, especially when found in the F3 generation [39, 40].

One limitation of the present study is the absence of baseline (pre-stress) CORT measurements, which precludes a clear distinction of within-subject differences between basal hormone levels and stress-induced reactivity. This limits the ability to determine whether the observed group differences in CORT reflect altered baseline HPA axis tone or differential responsiveness to stress. However, all animals were housed, handled, and tested under closely controlled, identical conditions, and blood sampling was conducted at consistent time points to minimize variability. Despite this limitation, the findings still offer valuable insight into group-level differences in HPA axis function under standardized conditions.

It is important to consider that the observed sex-dependent effects of F0 prenatal maternal stress on F3 offspring may also be shaped by alternative or interacting mechanisms beyond transgenerational transmission alone. Specifically, inherent sex differences in neurodevelopmental trajectories, baseline hormone levels, and stress reactivity are well-documented factors influencing behavioural and physiological outcomes including the HPA axis function [87]. For instance, the sex-specific patterns of HPA axis maturation and differential sensitivity to glucocorticoids during critical periods of development can modulate stress responsiveness in a way that is not solely attributable to ancestral stress exposures [88]. Furthermore, genetic heterogeneity, especially when using outbred strains, may contribute to variability in stress-related phenotypes and could interact with sex [89] to influence susceptibility or adaptability to early life stressors [90]. While these variables were not the primary focus of the present study, we recognize their potential relevance and suggest that future research incorporating controlled genetic backgrounds and longitudinal hormonal profiling could help disentangle these interacting variables.

## Conclusion

The present study shows that maternal gestational stress can be transmitted across three generations, leading to increased vulnerability to neuroimmune challenges and depressive-like behaviours particularly in females, in association with HPA axis dysregulation and psychomotor retardation in a sex- and task-specific manner in the unexposed F3 offspring. The findings support earlier accounts that CORT-independent mechanisms may contribute to stress-induced emotionality in rodents [42], because even in the absence of elevated CORT, psychomotor alterations may still be observed, possibly through non-adrenocorticotropin-mediated mechanisms [91]. Indeed, these findings provide new important insights for future investigation into stress-related psychomotor pathologies and related endocrine markers. Transgenerational stress generated new phenotypes in remote F3 descendants. While males may be more susceptible than females to the effects of immediate stress [92], across generations females will show heightened vulnerability to the compounding effects of generational stress. This phenotype must not be explained merely based on HPA axis dysregulation although CORT might still be causally involved in some aspects of their behaviour and physiology. The F3 generation represents the first generation in the female lineage that was not directly exposed to stress in the pregnant F0 generation, hence the present findings unequivocally suggest epigenetic inheritance of behavioural and physiological traits. Thus, influenced by ancestral maternal adversity, stress resilience and stress sensitivity in remote offspring follows a line of sex-dependent biobehavioural patterns, which are potentially linked to heritable epigenetic markers as embodied memories of stress [93, 94].

One of the most compelling avenues through which ancestral prenatal stress exerts long-lasting and sex-specific effects on neuroendocrine and immune outcomes is via epigenetic modifications, which shape the activity of stress-responsive systems such as the HPA axis [41, 43]. For example, epigenetic alterations in the promoter regions of key regulatory genes such as nuclear receptor subfamily 3 group C member 1 (*Nr3c1*) (encoding the glucocorticoid receptor) and *Crh* (encoding corticotropin-releasing hormone) have been shown to modulate glucocorticoid sensitivity and feedback inhibition of the HPA axis [95, 96], thus influencing stress reactivity across generations. These modifications are not only initiated by direct exposure to stress but potentially inherited via germline transmission [6]. Importantly, epigenetic marks are responsive to sex hormones and can thus underpin the observed sex-specific outcomes in stress responsivity and immune function [97]. Hence, in the context of ancestral maternal adversity, differential epigenetic programming may lead to enduring changes in HPA axis dynamics and cytokine expression patterns, with male offspring displaying heightened vulnerability.

Epigenetic regulation also plays a pivotal role in mediating the sexually dimorphic immune responses observed following ancestral stress exposure, particularly through its influence on cytokine gene expression. Stress-induced alterations in DNA methylation and histone modifications at promoter regions of cytokines such as IL-1β and IL-10 have been shown to follow sex-specific trajectories, likely due to differential sensitivity to sex hormones such as oestrogen and testosterone [98, 99]. In females, oestrogen receptor signalling interacts with epigenetic modifiers such as DNA methyltransferases and histone acetyltransferases, resulting in an enhanced transcriptional profile for anti-inflammatory genes like IL-10 [100, 101]. Conversely, males tend to exhibit stress-induced hypermethylation of anti-inflammatory genes and hypomethylation of pro-inflammatory loci, contributing to a more pronounced peripheral inflammatory state [102, 103]. These epigenetic modifications are influenced by early life adversity, including maternal stress, which can shape immune function in a lineage-specific manner through both germline and somatic transmission [68]. Moreover, recent evidence suggests that histone modifications such as increased H3K27 acetylation at IL-1β enhancers occur preferentially in females following early stress exposure [104], possibly underpinning the paradoxical upregulation of both IL-1β and IL-10 observed in F3 stressed females. The sex-biased epigenetic programming likely reflects a dynamic integration of ancestral stress, hormonal regulation, and immune signalling that may buffer or exacerbate vulnerability to stress-related neuroinflammatory disorders across generations. This also highlights the necessity of incorporating epigenetic frameworks to understand the persistent and intergenerational impact of early life stressors on physiological regulation and disease susceptibility. Future studies might benefit from an array of multiple assessments combined with an epigenetic approach to better understand the complex interactions between intrinsic developmental dynamics and extrinsic environmental adversities.

## Materials and methods

### Animals

This study involved four generations of female and male Long-Evans rats (*n* = 330 in total) bred in-house at the Canadian Centre for Behavioural Neuroscience and raised under closely controlled conditions. All rats were derived from a local breeding colony to avoid transportation stress. Rats were maintained on a 12-h light/dark cycle (lights on at 7:30) with *ad libitum* access to food and water. Timed pregnancy was achieved by pairing dams with a male for 1 h per day until mating occurred. Pregnancy of the dams was confirmed by weight gain 10–12 days later. All rats were housed in same-sex pairs except for pregnant rats, which were housed individually from gestational day 19 until weaning of the offspring.

Prior to behavioural testing, adult rats (12–18 weeks old) were handled for approximately 5 min daily for 2–3 consecutive days for habituation to the experimenters. The experimenters were blinded to the experimental treatments. Body weight was monitored throughout the experiment. All testing and training were performed during the light phase of the cycle at the same time of day. Parental generations were also housed under standard conditions in same-sex pairs with *ad libitum* access to food and water following the same protocol for housing and handling. All experimental procedures were performed under protocols approved by the Animal Care Committee of the University of Lethbridge in compliance with the guidelines of the Canadian Council on Animal Care.

### Study design

**Figure 6.**
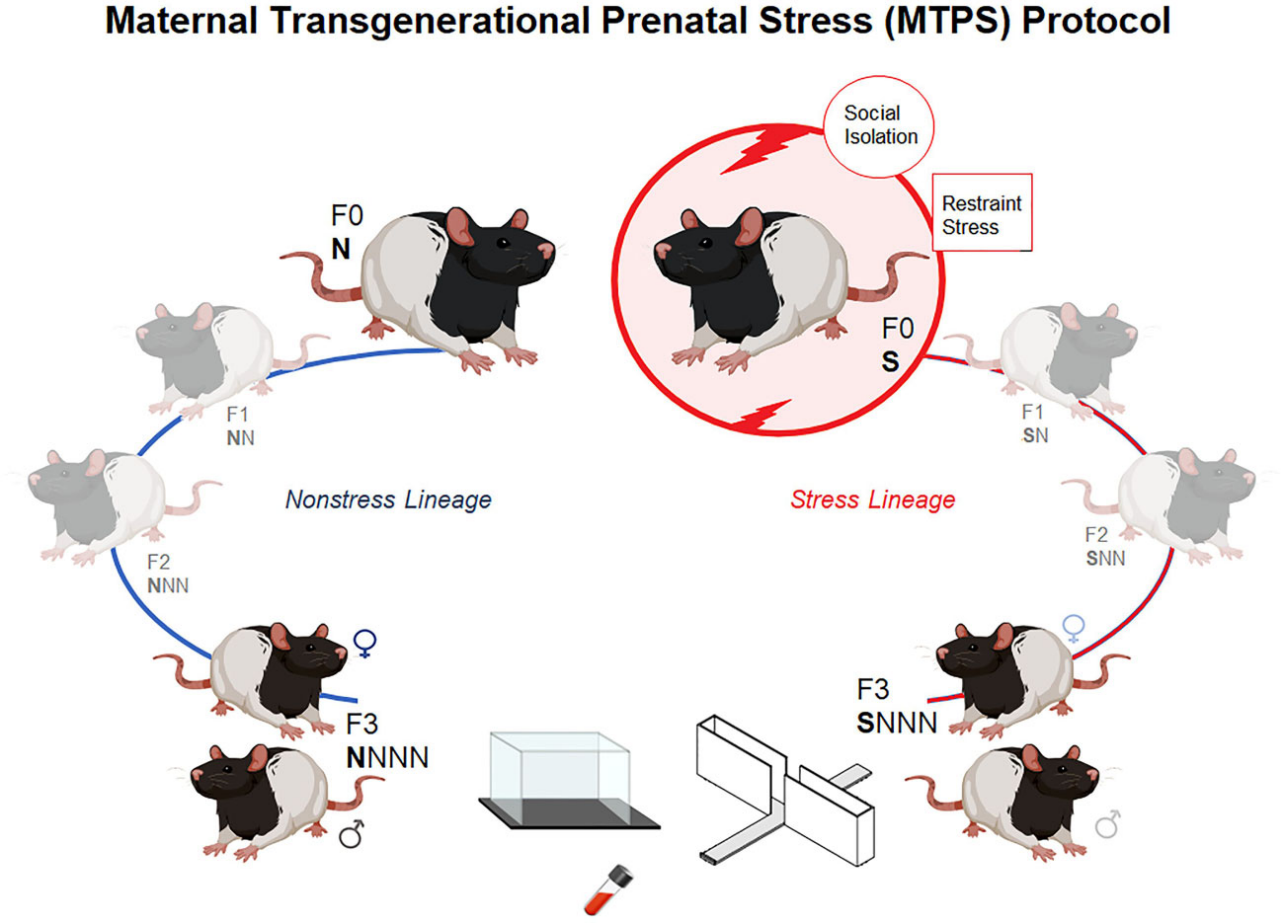
Experimental design of the maternal transgenerational prenatal stress (MTPS) protocol in rats. The flow chart illustrates that F0 pregnant dams were subjected to a psycho-social stress (social isolation and restraint stress) from gestational days (GD) 12 to 18. Their pregnant daughters (F1; SN), grand-daughters (F2; SNN), and great-grand-daughters (F3; SNNN) remained undisturbed in order to generate a transgenerational maternal stress lineage. The F1–F3 females were bred with non-stressed males. An alternative lineage of non-stressed mothers (non-stressed F0–F3) were also used as controls (F3; NNNN). Accordingly, the F1–F3 offspring in the stressed lineage and F0–F3 offspring from the non-stressed lineage were left unstressed throughout the experiment. F3 females and males from both lineages were examined in the present study for behavioural changes in the open field and elevated plus maze along with blood sample collection. *S*: stressed, *N*: non-stressed, *F*: filial generation. *Diagram created with Biorender.com*.

The experimental design (Fig. 6) in the present study involved exposing pregnant rat dams of the parental generation (filial 0; F0) to psychosocial stress during gestation, whereas three generations of their female offspring (F1–F3 generations) were bred with non-stressed control males and did not undergo gestational stress. Briefly, F1 female offspring (S**N**) born to stressed F0 dams (**S**) were bred to produce the subsequent F2 (SN**N**) generation. The F2 SN**N** female offspring were again bred to yield the F3 generation (SNN**N**). Accordingly, the present cohort generated an MTPS lineage in which only the timed-pregnant parental F0 dams were subjected to stress. F3 rats born to the SNN**N** lineage were compared to F3 offspring generated from a non-stressed control lineage (NNN**N**) [6]. These controls were born to a lineage of non-stressed F0 mothers (**N**). Prior to testing, F3 animals from the NNN**N** and SNN**N** lineages were assigned to one of the following experimental groups: (1) female control (*n* = 90), (2) female stress (*n* = 85), (3) male control (*n* = 67), and (4) male stress (*n* = 88).

### Psychosocial stress protocol

Timed-pregnant F0 female rats underwent periods of restraint and SI stress during mid-late gestation [gestation days (GD) 12–18]. Stress procedures were implemented at different times and days in a semirandom sequence to avoid habituation to the stressor [8]. For the restraint protocol, animals were placed in a customized transparent plexiglass container for 15–60 min in the morning or evening. The container was placed vertically and adjusted to the animals’ size to prevent them from turning but without compressing their body. The F0 animals were also subjected to 17 h of overnight SI stress on GD14 and GD17, in which they were housed alone from 16:00 to 09:00 h of the following morning but could still hear and smell their counterparts. The F1–F3 offspring in the stressed lineage were left unstressed throughout the experiment. Moreover, non-stressed F0–F3 generations served as matched controls that were bred alongside the stressed lineage.

### Open field activity

Locomotor activity and exploratory behaviour was assessed in an automated open field apparatus according to the method described by Faraji et al. [105]. Briefly, an AccuScan activity monitoring system (AccuScan Instruments Inc., OH, USA) connected to clear Plexiglas boxes (length 42 cm, width 42 cm, and height 30 cm) was used to assess exploratory activity and affective states in an open field environment. The setup allowed to monitor and assess 10 animals in 10 boxes simultaneously. Animals were placed individually into an open field and monitored for 10 min. Total travel distance, movement time, rest time, margin distance, centre distance, number of rearings, and rearing time were analysed using VersaDat^TM^ software (AccuScan Instruments Inc., OH, USA) to measure overall activity. After each animal, the apparatus was thoroughly cleaned with 10% Clinicide (Vetoquinol, QC, Canada) to eliminate any odour traces.

### Elevated plus maze

Anxiety-like behaviour was assessed using an EPM made of black Plexiglas [28]. The apparatus consisted of two open and two closed arms (each 40 long × 10 cm wide) and was elevated 90 cm above the floor. The open arms had no side or end walls, whereas the closed arms featured side and end walls (40 cm high). Rats were placed individually in the central square (10 cm × 10 cm) facing either the left or right open arm and were allowed to explore the maze for 5 min while being video recorded from a dorsal perspective. The experimenter left the room immediately after placing the rat on the maze to reduce confounding effects of human presence [81]. Five standard measures of the EPM (total time spent in open arms and in the ends of open arms, total time spent in closed arms, total time spent in centre square, and total time spent risk assessing) were evaluated by an experimenter blind to the experimental groups. In order to minimize olfactory cues, after each animal the apparatus was cleaned with 10% Clinicide (Vetoquinol, QC, Canada).

### Blood samples collection and analysis of CORT and cytokines

All rats in this study underwent blood sampling following behavioural assessments, and the procedure for blood sampling was the same as previously reported [106]. Briefly, rats were transported individually to the surgical suite and anesthetized with 4% isoflurane. During 2–3 min of anaesthesia, 0.5–0.7 ml of blood was collected in the morning hours (9:00***–***11:00 a.m.) by two experimenters from the tail vein using a heparinized butterfly catheter. Blood was then transferred to centrifuge tubes and plasma was obtained after centrifugation at 5000 rpm for 5 min. The plasma samples were stored at −20°C until analysed for CORT concentration using commercial radioimmunoassay kits (Abcam Inc., Toronto, ON, Canada). Also, a custom 5-plex rat assay of pro- and anti-inflammatory cytokines (interleukins IL-1β and IL-10), and VEGF were simultaneously measured in samples using Eve Technologies' Custom Rat Cytokine 5-Plex (MilliporeSigma, Burlington, MA, USA) according to the manufacturer’s protocol. Assay sensitivities of these markers ranged from 1.9 to 30.7. Individual analyte sensitivity values are available in the MilliporeSigma MILLIPLEX^®^ MAP protocol. The multiplexing analysis was performed using the Luminex™ 200 system (Luminex, Austin, TX, USA) by Eve Technologies Corp. (Calgary, AB, Canada). No behavioural testing was performed on blood sampling days.

### Statistical analysis

The data in the present study were normally distributed and Levene’s test confirmed homogeneity of variance in the data. All data were analysed using an SPSS 21.0 package (SPSS Inc., Armonk, NY, USA). Effects of main factors (group; four levels and sex; two levels) were analysed for the behavioural measures in the open field activity box and EPM by one-way and multivariate ANOVA, and Pearson’s correlation coefficient. Tukey’s HSD test was used to control for family-wise error in *post hoc* comparisons following ANOVA, and the number of overall tests was limited to reduce the risk of inflated Type I error. Linear regression analysis was also performed to predict the outcome (dependent) variables (exploratory and anxiety-like behaviours) through the predictor (independent) variable (CORT levels). The assumptions underlying regression analyses including linearity, normality of distribution, and the absence of outliers were assessed using diagnostic plots (Q-Q plots) by SPSS and found to be adequately met. Because cytokine values were not normally distributed, and Levene’s test did not confirm homogeneity of variance in IL-10, Kruskal–Wallis *H* test, a rank-based nonparametric test for analysis of variance was used to detect differences between means of groups. Following significant Kruskal–Wallis tests, Dunn’s *post hoc* tests with Holm–Bonferroni correction were used to adjust for multiple comparisons. A *P*-value of *<*.05 was considered statistically significant. Results are presented as mean ± SEM.

## Data Availability

Supplementary data are available at EnvEpig online. Further data are available upon request from the study authors.
